# Development, Evaluation, and Molecular Docking of Oral Dissolving Film of Atenolol

**DOI:** 10.3390/pharmaceutics13101727

**Published:** 2021-10-19

**Authors:** Karina Citra Rani, Nani Parfati, Ni Luh Dewi Aryani, Agnes Nuniek Winantari, Endang Wahyu Fitriani, Aditya Trias Pradana, Roisah Nawatila, Astridani Rizky Putranti, Florencia Irine, Florentia Angelica, Cintya Yohanes, Christina Avanti

**Affiliations:** 1 Department of Pharmaceutics, Faculty of Pharmacy, University of Surabaya, Surabaya 60293, Indonesia; karinacitrarani@staff.ubaya.ac.id (K.C.R.); nani_parfati@staff.ubaya.ac.id (N.P.); dewi_aryani@staff.ubaya.ac.id (N.L.D.A.); agnes_nuniek@staff.ubaya.ac.id (A.N.W.); endangwahyu@staff.ubaya.ac.id (E.W.F.); aditya_trias@staff.ubaya.ac.id (A.T.P.); roisah@staff.ubaya.ac.id (R.N.); astridaniputranti@staff.ubaya.ac.id (A.R.P.); florenirine21@gmail.com (F.I.); florentiaangelica@gmail.com (F.A.); cintya.yohanes@yahoo.co.id (C.Y.); 2 Department of Pharmaceutics and Industrial Pharmacy, Faculty of Pharmaceutical Sciences, Chulalongkorn University, Bangkok 10330, Thailand

**Keywords:** oral dissolving film, atenolol, HPMC E5, CMC-Na, Na-alginate

## Abstract

The development of oral dissolving film (ODF) of atenolol is an attempt to enhance convenience and compliance for geriatric patients suffering from hypertension. Film former is the most essential component in ODF that determines the physical characteristic and drug release. In this study, three different types of film former including HPMC E5 4% (*w*/*v*), 5% (*w*/*v*), CMC-Na 3% (*w*/*v*), 4% (*w*/*v*), and Na-alginate 2.5% (*w*/*v*), 3% (*w*/*v*) were optimized in Formula 1 (F1) to Formula 6 (F6), respectively. A solvent casting method was employed to develop ODF of atenolol. The films formed by HPMC E5 produced a smooth and flexible surface, whereas CMC-Na and Na-alginate produced gritty textured films. Satisfactory results were obtained from several physical parameters such as film thickness, folding endurance, swelling index, and disintegration time. The homogeneity, drug content, and dissolution properties of ODF with HPMC exhibited better characteristics than the other formulas. Formula 1 exhibited the highest drug release compared to the other ODFs. The molecular docking results showed that there was a hydrogen bonding between atenolol and film formers which was also supported by the FTIR spectrum. The findings of this study suggest that HPMC E5 is the most favorable film former for ODF of atenolol.

## 1. Introduction

The most preferred route for drug administration is the oral route. The development of an innovative drug delivery system to enhance patient convenience and compliance is needed. Oral dissolving film (ODF) is an invention to provide convenience to patients, especially for geriatric patients suffering from dysphagia [1]. ODF is stable in the solid film-form but easily disintegrated upon contact with saliva in the oral cavity. This dosage form does not require water for administration and gives quick absorption [2]. ODF also provides another advantage for highly permeable drugs of which the first-pass effect is significant for those drugs [3]. Formulation of these drugs in ODF facilitates the drug release through the buccal mucosa for direct absorption. This mechanism can reduce the extent of the first-pass effect and food interactions during the absorption process [4]. ODF may also exhibit a faster pharmacological response due to the faster disintegration, dissolution, and trans-mucosal absorption process [5].

Atenolol is an antihypertension drug with a relatively polar hydrophilic compound and a water solubility of 26.5 mg/mL. The oral bioavailability of atenolol is approximately 45–55%, due to the first-pass effect mechanism [5]. This drug is also widely prescribed as first-step therapy for hypertension in geriatric patients who have difficulty swallowing. Therefore, the development of ODF atenolol is a promising strategy to enhance the convenience and therapeutic efficacy of atenolol. Hence, the present study aims to formulate oral dissolving films of atenolol that produce desired physical and drug release characteristics. The most essential component for ODF preparation is a polymer, which serves as a film former. The type and concentration of film formers influence the robustness, disintegration, and drug release characteristics of ODF [3]. Various hydrophilic polymers that facilitate rapid dissolution, desirable mechanical properties, good mouthfeel properties, and low water vapor absorption are suitable candidates for a film former [6]. 

Hydroxypropyl methylcellulose (HPMC) E5, carboxymethyl cellulose sodium (CMC-Na), and sodium alginate (Na-alginate) are several polymers that have film-forming abilities [7]. HPMC E5 revealed excellent flexibility and uniformity in the development of ODF. Moreover, HPMC E5 promotes the dissolution rate of flupentixol, which is incorporated in these films [8]. CMC-Na also exhibits suitable characteristics of oral thin film development. The study of probiotic dissolving films to prevent dental caries was conducted using CMC-Na as film former. The obtained oral thin film is characterized as a uniform film with excellent thickness, dissolving time, hygroscopicity, and biotherapeutic properties [9]. Films with a 2–5% *w*/*v* concentration of both polymers (HPMC E5 and CMC-Na) are clear, transparent, and of preferable mechanical characteristics [8]. Sodium alginate also served as a desirable film former which promotes drug release through swelling mechanism [10]. Sodium alginate also has good film properties and is non-sticky when applied as film former [11]. The concentration of sodium alginate in film formulation was 1.5–3% *w*/*v* [12]. 

In this study, three different types of film former (HPMC E5, CMC-Na, and Na- alginate) were optimized to produce ODFs and six formulas of HPMC E5 4% (*w*/*v*) (F1); HPMC E5 5% (*w*/*v*) (F2); CMC-Na 3% (*w*/*v*) (F3); CMC-Na 4% (*w*/*v*) (F4); Na- alginate 2.5% (*w*/*v*) (F5); Na- alginate 3% (*w*/*v*) (F6) were developed. All six formulas were chosen based on our preliminary study to find an optimum gel former concentration that produced ODF with acceptable characteristics, such as being thin, flexible, and having adequate mechanical properties. The solvent casting method was used to develop all of these formulas as the method is the most visible method in the optimization study of pharmaceutical ODF. In fact, this method can be easily applied, and sophisticated equipment is not required. All of the film components were suspended or dissolved in water, then the dispersion or solution was quickly cast and dried to produce a film [12]. The effect of film former type and concentration were evaluated to the physical characteristics of ODFs such as film thickness, folding endurance, swelling index, and disintegration time [11]. Drug content and in vitro drug dissolution were also compared among these formulas [13]. IR spectrum study and molecular docking were also conducted to analyze the interaction between atenolol and film former. Morphological characteristic of ODFs was also observed using a scanning electron microscope. Those evaluations were conducted to obtain the most desirable ODF of atenolol. 

## 2. Materials and Methods

### 2.1. Materials

Atenolol and Poloxamer 188 were kindly supplied by PT. Kalbe Farma Tbk. by stating that Atenolol pharmaceutical grade was purchased from Refarmed Chemicals Ltd. (Lugano, Switzerland) and Poloxamer 188 pharmaceutical grade was purchased from BASF Corporation (Ludwigshafen, Germany). Hydroxypropyl methyl cellulose (HPMC) E5 pharmaceutical grade was purchased from Wuhan Senwayer Century Chemical Co., Ltd. (Wuhan, China). Sodium carboxymethyl cellulose (CMC-Na) pharmaceutical grade was purchased from Handan Yaxiang Chemical (Handan, China), Sodium alginate (Na-alginate) pharmaceutical grade (I-3 type) was purchased from Kimica Corporation (Tokyo, Japan). Glycerin pharmaceutical grade was purchased from PT. Wilmar Nabati Indonesia (Gresik, Indonesia). Citric acid, potassium acesulfame, methyl paraben, and orange flavor pharmaceutical grade were purchased from Merck and Co. Inc. (Kenilworth, IL, USA). Propylene glycol pharmaceutical grade was purchased from Shell Chemicals (The Hague, The Netherlands). All other chemicals were of analytical grade such as sodium dihydrogen phosphate was purchased from Merck KGaA (Darmstadt, Germany), disodium hydrogen phosphate was purchased from Merck KGaA (Darmstadt, Germany), dibutylamine was purchased from Merck KGaA (Darmstadt, Germany), glacial acetic acid was purchased from Merck KGaA (Darmstadt, Germany), and sodium1- heptane sulfonate was purchased from Tokyo Chemical Industry Co. Ltd (Tokyo, Japan). 

### 2.2. Methods

#### 2.2.1. Sample Preparation

Three different types of film former include HPMC E5 4%, 5% (*w*/*v*); CMC-Na 3%, 4% (*w*/*v*); and Na- alginate 2.5%, 3% (*w*/*v*) were optimized in Formula 1 (F1) to Formula 6 (F6), respectively, as exhibited in Table 1. The solvent casting method was used to develop ODF of atenolol.

A polymeric solution was prepared by dissolving the film-former polymer in a specific proportion in an excipient mixture consisting of potassium acesulfame, citric acid, poloxamer 188, orange flavor, and sunset yellow FDC in purified water and methyl paraben in propylene glycol. This mixture was maintained at 2–2.5 h. After that, glycerin was added to the atenolol powder so that it could be wetted. Then, this solid mixture was added to the polymeric solution gradually and stirred for 45 min at 800 rpm and then left until the air bubbles disappeared (without sonication). The solution was cast onto an acrylic plate (18 × 18 cm) and then dried at 50 ± 2 °C for 15 h. The resultant film was cut to a size of 2 × 3 cm, in which 25 mg of atenolol was included. Films having air bubbles, cuts, or imperfections were excluded from the study. The next step is to evaluate the films.

#### 2.2.2. Film Thickness

The thickness of the film was normally measured at five different locations of the films using a micrometer screw gauge. The standard deviation and mean value were then calculated. The ideal film thickness ranges from 50–1000 µm [14]. The other study also posits that ODF thickness below 0.3 mm is still acceptable [15]. 

#### 2.2.3. Folding Endurance

To determine the folding endurance, a strip of film was repeatedly folded at an angle of 180° at the same place until it broke. Films with a folding endurance value of 300 or more are considered to be ideal [14]. A higher folding endurance value signifies the more mechanical strength of the film [16].

#### 2.2.4. Swelling Index

A simulated saliva solution was used to check the swelling studies of films. This method required a petri dish, plate glass, and digital analytic scale where the film was then weighed and immersed into simulated saliva solution (phosphate buffer pH 6.8). An increase in the weight of the film is noted as constant predetermined time intervals until no more increase in weight [16]. Then, the swelling index can be determined by these parameters:

$$\mathrm{Swelling}\;\mathrm{index}=\frac{w_{t}-{\;w}_{0}}{w_{0}}$$

w_t_ = weight of film at time interval t (final weight)
w_0_ = weight of film at time 0 (initial weight).

#### 2.2.5. Determination of Disintegration Time

The disintegration time test was conducted manually by placing the film into a 10 mL simulated saliva solution (phosphate buffer pH 6.8) in a beaker glass and stirred continuously every 10 s. The time required for the film to dissolve completely is considered as the disintegration time. Ideally, the disintegration time for an ODF ranges from 5–30 s, though disintegration within 60 s is acceptable [16].

#### 2.2.6. Weight Uniformity

The weight uniformity test was carried out by weighing 20 units of ODF at a size of 2 × 3 cm. The weight of the ODF were tabulated and the results were expressed as the average weight ± SD. 

#### 2.2.7. Drug Content

The drug content in a film is determined by a standard assay method specified for individual drug in different pharmacopoeia. ODF was dissolved with 50 mL mobile phases in a glass beaker and sonicated for 5 min. The dissolved film was taken into a 100 mL volumetric flask and dissolved in mobile phase (100 mL). Then, 5 mL of this solution was taken into a 50 mL volumetric flask and dissolved in the mobile phase (50 mL). Afterward, it was filtered using a 0.5 µm membrane filter, and the drug content was observed using the UPLC H Class Waters (Milford, MA, USA), equipped with Empower 3 software as data system [17]. The stationary phase used was Acquity UPLC BEH C18 1.7 µm (2.1 × 50 mm column) and the detector was a UV detector.

Preparation of the mobile phase was used 1.1 g of sodium 1-heptansulfonate and 0.71 g of sodium phosphate and dissolved in 700 mL of purified water. Then, 2 mL of dibutyl amine and phosphoric acid 0.8 M were added to the mixture until it reached pH 3.0. Finally, 300 mL methanol was added to the mixture. Then, it was filtered using a 0.5 µm membrane filter [17]. The flow rate for the mobile phase was 0.3 mL/min. 

#### 2.2.8. In Vitro Drug Dissolution

In vitro drug dissolution study (*n* = 4) were performed utilizing a Hanson SR8-Plus dissolution test system (Hanson Research Co., Chatsworth, CA, USA) according to USP 40 apparatus 5, paddle over disk method. The dissolution medium used was 900 mL of acetate buffer pH 4.6. The temperature was maintained at 37 ± 0.5 °C and rotation speed of 50 rpm was repeatedly adjusted. Sample of drug dissolved was collected at predetermined intervals and was determined by using the ULPC method [17].

#### 2.2.9. Surface pH

Surface pH was determined to evaluate the acceptability of the ODF administration in the oral cavity. ODF was placed in a petri dish, then wetted by using 0.5 mL purified water for 60 s. The pH of ODF was measured by touching a pH meter (Schott pH meter LAB 850, Mainz, Germany) electrode of with the ODF surface. The surface pH of ODF should be within the normal range of salivary pH 6.8–7.4 [5]. 

#### 2.2.10. Film Morphology Using Scanning Electron Microscope (SEM)

Morphological analysis of the film was performed using a scanning electron microscope (Hitachi High Technologies America, Inc., Schaumburg, IL, USA). The film was directly fixed on a metal stub using double side adhesive tape. Samples were gold-coated at 10 mA for 20 s. Surface morphology can be obtained at 15 kV and 5 kV, respectively at 1000 and 5000 times magnification [18].

#### 2.2.11. IR Spectrum Study 

Fourier transform infrared (FT-IR) studies were carried out to analyze the possible drug-excipient interaction. The FT-IR spectrum of pure drug, film former, and extruded films were recorded using an FT-IR spectrometer (Shimadzu FT-IR 4200, Kyoto, Japan) over a scan range from 4000 to 400 cm^−1^. The FT-IR spectrum of the atenolol (pure drug), film former (HPMC, CMC-Na, and Na-alginate), and oral dissolving films were compared to determine drug-excipient interaction, especially film former [10].

#### 2.2.12. Molecular Docking Study

The two and three-dimensional (3-D) structures of atenolol and film-forming agents were constructed using MarvinSketch program version 19.20 software (ForOSX), ChemAxon Ltd., Budapest, Hungary. Similar software was utilized for energy minimization of structures of the molecules. Prior to the study, the International Union of Pure and Applied Chemistry (IUPAC) name and the chemical structure of atenolol and film-forming agents were confirmed using PubChem^®^. The interactions between atenolol and film-forming agents were determined in silico by molecular docking using Autodock 4.2 software. The grid box covered all the molecules and spacing (Armstrong) was 0.375. The free binding energy (∆G) in silico of atenolol-sodium alginate, atenolol-CMC Na, and atenolol-HPMC, were obtained after running 100 times of the molecular docking by Autodock 4.2. The bondings and the interactions distances between atenolol and film-forming agents. The Discovery Studio Visualizer (DSV), Biovia, Dassault system, San Diego, CA, USA) was utilized for visualizing the bonding and the interactions distances between atenolol and film-forming agents.

#### 2.2.13. Data Analysis

The results of physical characteristics evaluation such as folding endurance, swelling index, and in vitro drug dissolution were analyzed using one-way ANOVA (*p* < 0.05). Other parameters including film thickness, weight uniformity, drug content, and surface pH were analyzed by comparing the results to the specified requirements. 

## 3. Results

### 3.1. Visual Appearance 

**Figure 1:**
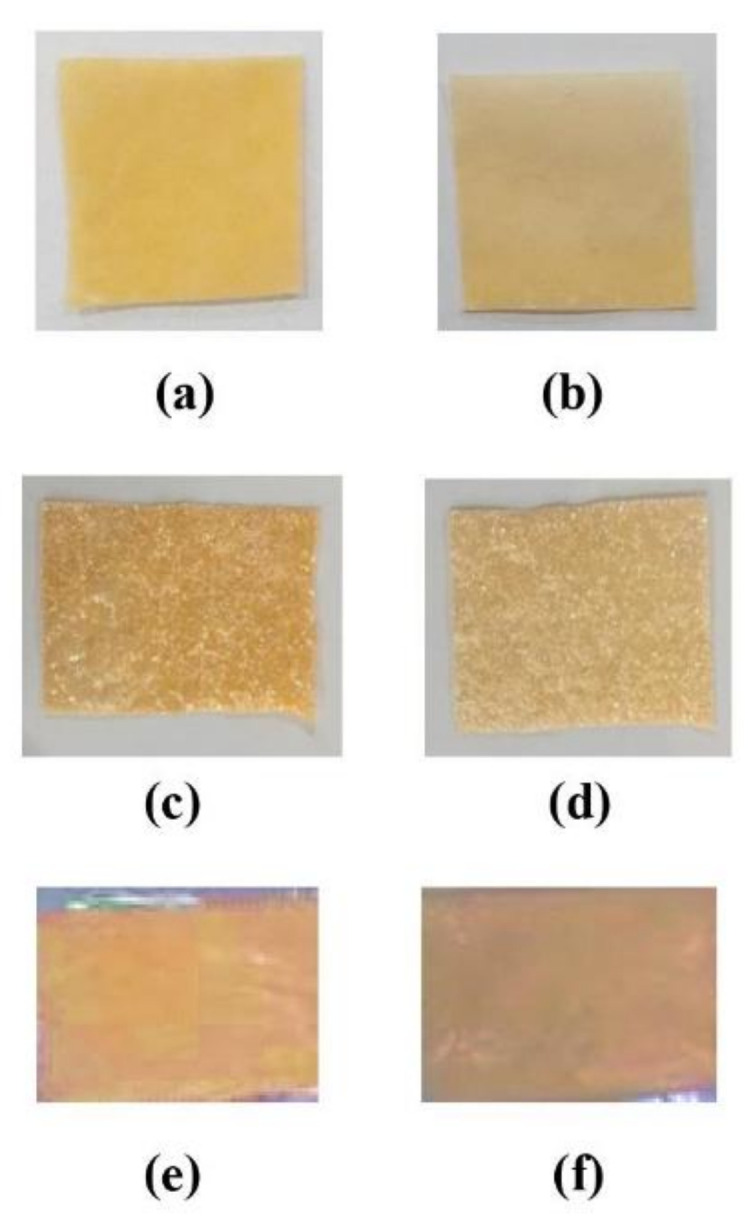
Physical appearance of the ODF. (**a**) F1 with 4% HPMC E5; (**b**) F2 with 5% HPMC E5; (**c**) F3 with 3% CMC-Na; (**d**) F4 with 4% CMC-Na; (**e**) F5 with 2.5% Na -alginate; and (**f**) F6 with 3% Na- alginate.

The visual observation results of the six ODF formulas are shown in Figure 1. From the organoleptic results, it can be seen that the majority of the formulas exhibited flexible film with a smooth surface. However, the ODF formulated from CMC-Na as a film former formed a gritty texture. Other organoleptic results were sweet with slightly bitter taste without any odor.

### 3.2. Film Thickness

Table 2 shows that HPMC E5 produced a higher film thickness compared to CMC-Na and Na-alginate. However, all of the film formers keep exhibited suitable film thickness of oral dissolving films.

### 3.3. Folding Endurance

**Figure 2:**
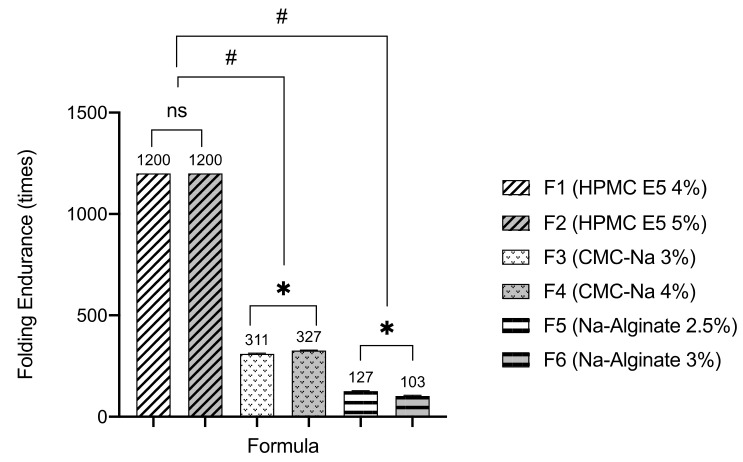
Folding endurance in times of the ODFs (mean ± SD, *n* = 4). The (*) indicates a significant different among similar polymer type groups (*p* < 0.05); the (#) indicates a significant different between different polymer type groups (*p* < 0.05); and the (ns) indicates a non-significant different (*p* > 0.05).

Observations of endurance on the folding process in the six formulas are presented in Figure 2. The ODF of atenolol formula using HPMC E5 and CMC-Na met the folding endurance requirement of ODF (>300 times), where F1 and F2 obtain excellent value. However, ODF prepared from Na-alginate produced a brittle film and exhibited a low value of endurance.

### 3.4. Swelling Index

**Figure 3:**
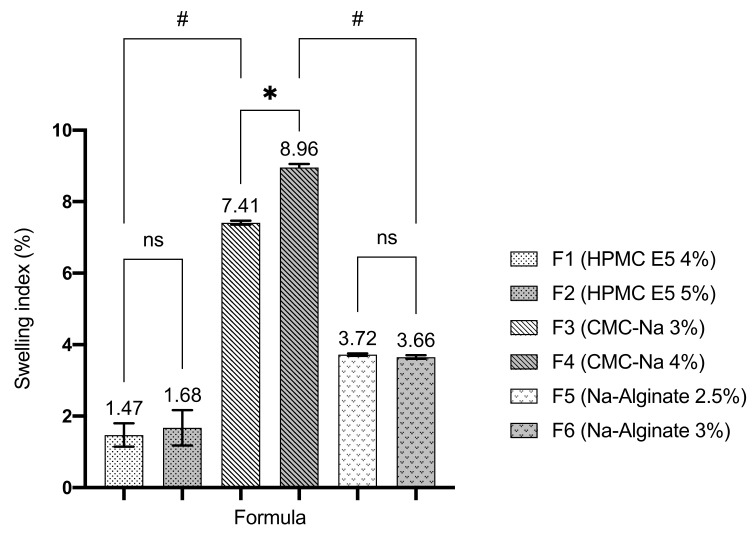
Swelling index of ODFs in simulated saliva solution (mean ± SD, *n* = 4). The (*) indicates a significant different among similar polymer type groups (*p* < 0.05); the (#) indicates a significant different between different polymer type groups (*p* < 0.05); and the (ns) indicates a non-significant different (*p* > 0.05).

The swelling index of ODF formulated with CMC-Na showed a significant value compared to the other two film formers, as seen in Figure 3. However, the increased concentration of each film former did not show a significant difference to the swelling index of the dosage forms.

### 3.5. Disintegration Time

Table 3 shows the disintegration time of the ODF in simulated saliva solution (phosphate buffer pH 6.8). F5 and F6 show significantly faster disintegration times compared to the other four formulas. Increasing the concentration of the film former also increases the time needed for ODF to disintegrate.

### 3.6. Weight Uniformity

**Figure 4:**
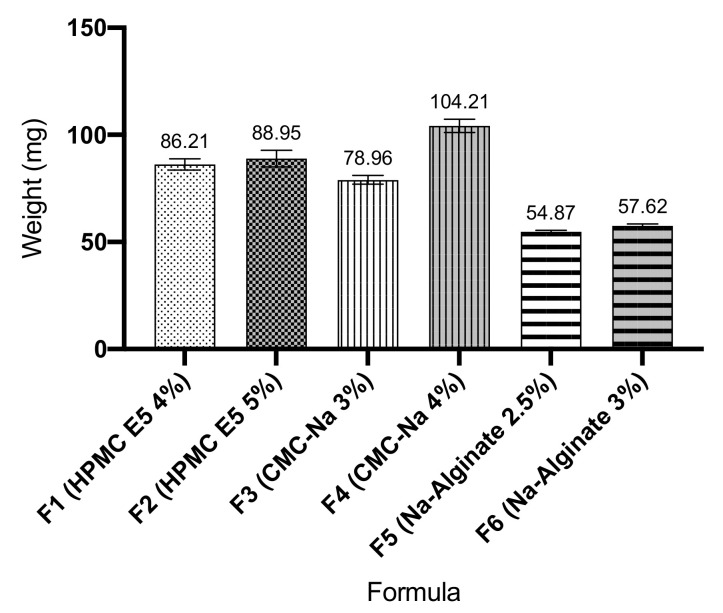
Oral dissolving films weight (mean ± SD, *n* = 4).

It can be seen in Figure 4 that an increase in the concentration of the film former in the formula resulted an in an increase in the weight of the formulated ODF. Furthermore, each prepared formula resulted in a good uniformity of weight with a low deviation between manufacturing replications.

### 3.7. Drug Content

**Figure 5:**
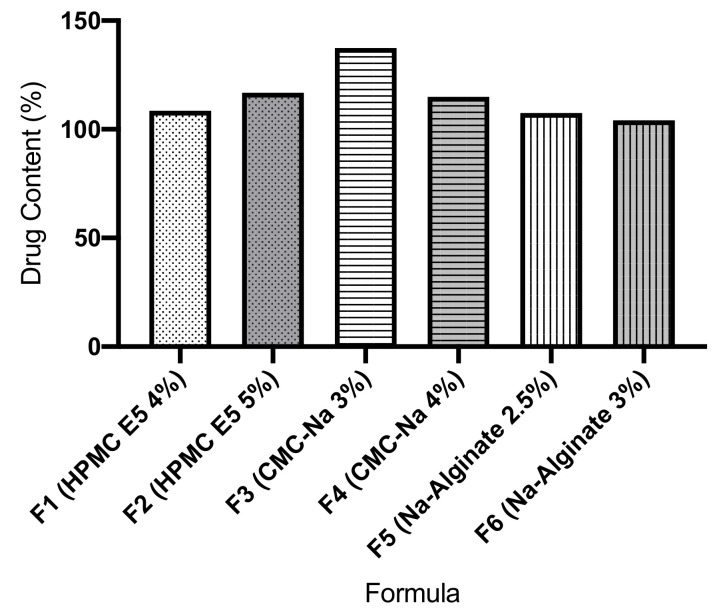
Atenolol content in oral dissolving films measured by UPLC method.

Atenolol content in ODF was measured by UPLC. The results of these observations can be seen in Figure 5, where the drug content is quite large in all formulas. The highest drug content is in Formula 3 with 3% CMC-Na as the film former, while the lowest was in Formula 6 with 3% Na-Alginate as the film former.

### 3.8. In-Vitro Drug Dissolution

**Figure 6:**
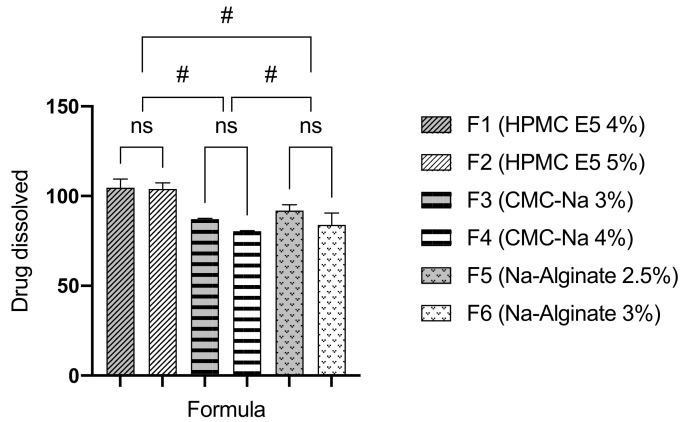
Percentage of atenolol dissolved from ODFs within 30 min of the dissolution test (mean ± SD, *n* = 4). The (#) indicates a significant different between various polymer type groups (*p* < 0.05); The (ns) indicates no significant different (*p* > 0.05).

The atenolol dissolution rate of ODFs was carried out in a 4.6 pH acetate buffer medium. Samples were taken after 30 min, and the amount of drug dissolved is shown in Figure 6. F1 and F2 (formulated from HPMC E5 as the film former) showed the highest percentage of drug dissolved. Meanwhile, increasing the concentration of the film former also decreases the dissolution rate of atenolol from ODFs.

### 3.9. Surface pH 

**Figure 7:**
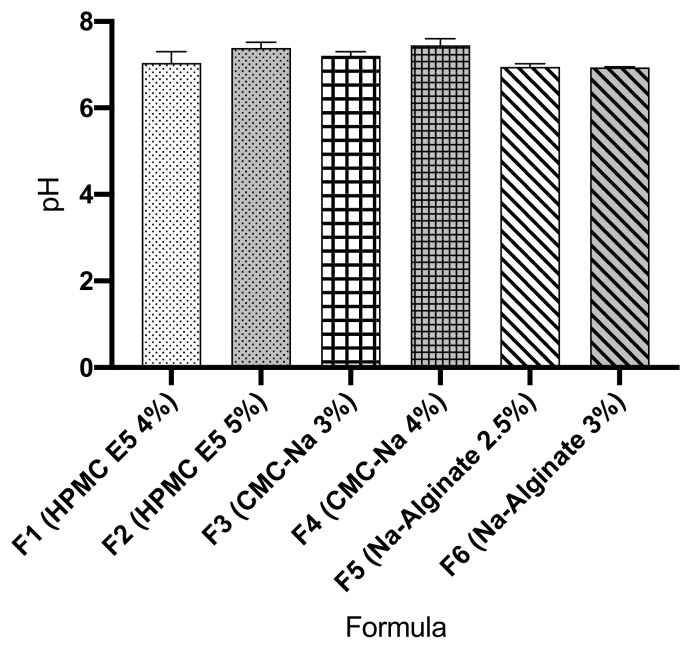
Surface pH of atenolol oral dissolving films.

The surface pH of the films must be determined because either the highly acidic or highly basic pH of ODF cause irritation and discomfort during administration. The surface pH of all the ODF formulas (F1 to F6) was is in the range of 6.95–7.45. The results were tabulated in Figure 7. ODF of atenolol produced in this study exhibited neutral pH, therefore these ODFs are safe and acceptable to be used in the oral cavity without any problem concerning irritation.

### 3.10. Particle Shape and Morphology

**Figure 8:**
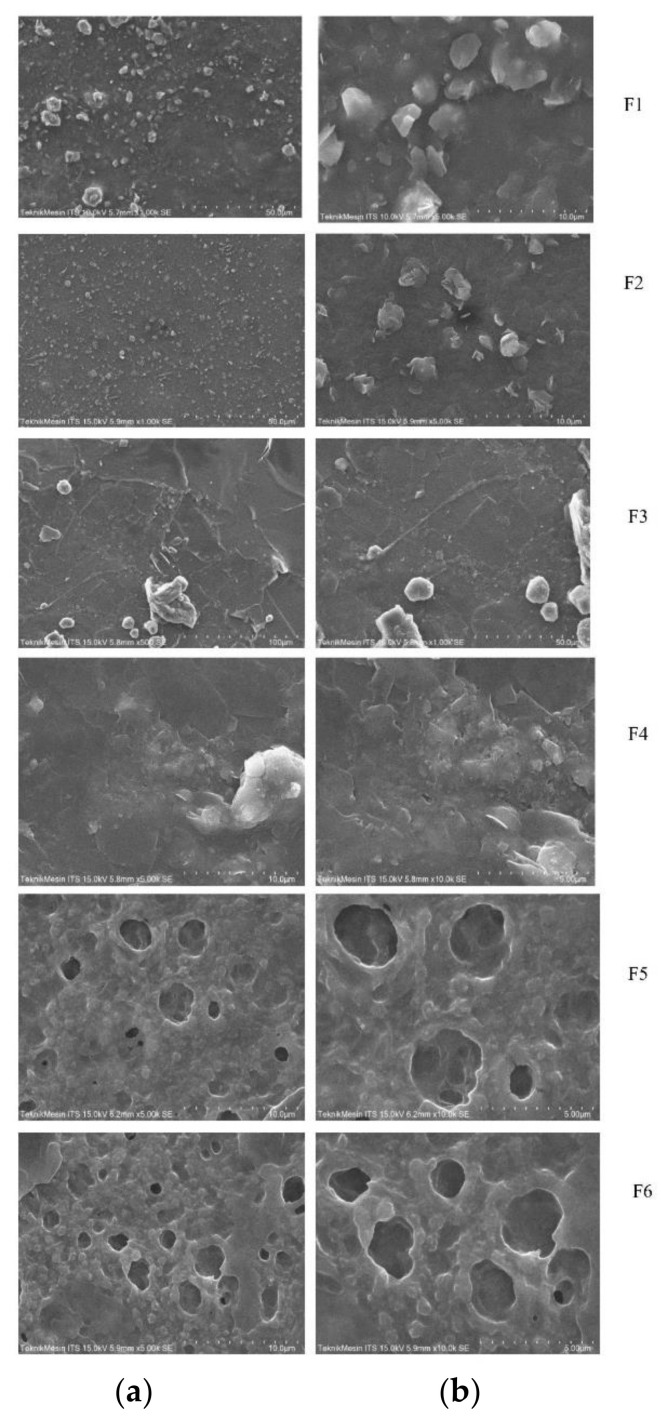
Scanning electron microscope image of atenolol ODF in (**a**) 1000× magnification and (**b**) 5000× magnification.

The particle shape and morphology of the ODFs were analyzed using a scanning electron microscope. Microscopic images of ODF are shown in Figure 8. Atenolol ODF formulated from HPMC E5 as film former (F1 and F2) showed gritty texture due to the deposition of drug crystals on the film surface. Small pores were also observed in the ODF structure. However, F3 and F4 (formulated from CMC-Na as a film former) exposed lamellar particles with small pores and rough surfaces. Meanwhile, F5 and F6 (formulated from Na- alginate as a film former) produced rough particles with wider pores.

### 3.11. FT-IR Spectrum Analysis

**Figure 9:**
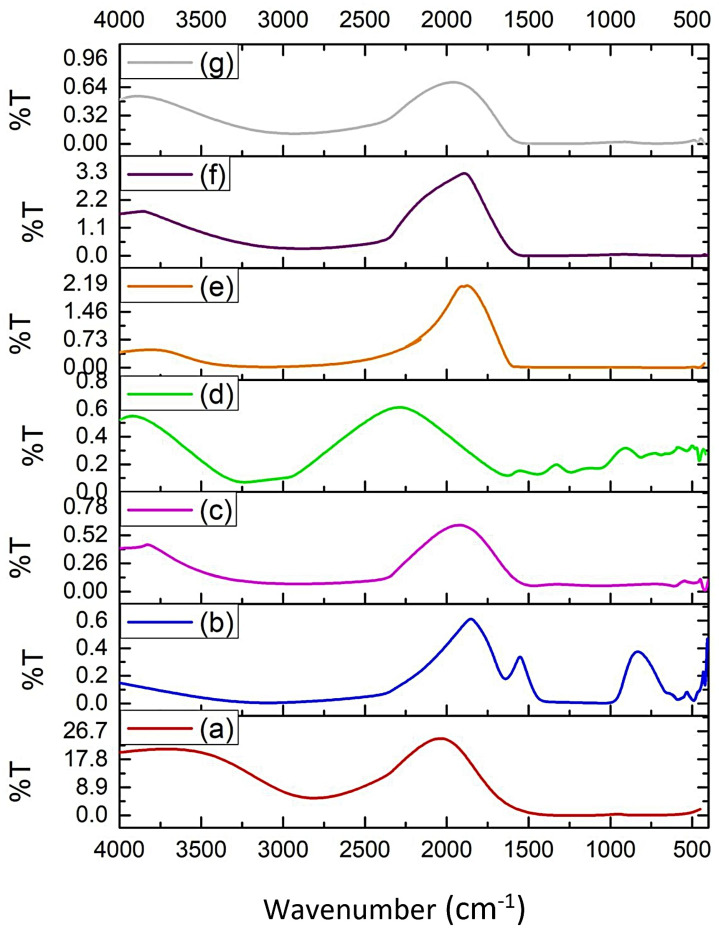
FT-IR spectrum of (**a**) Atenolol; (**b**) HPMC E5; (**c**) CMC-Na; (**d**) Na-alginate; (**e**) ODF produced from HPMC E5 as film former; (**f**) ODF produced from CMC-Na as film former; and (**g**) ODF produced from Na-alginate as film former.

The results of FT-IR spectrum analysis of atenolol, polymer as film former (HPMC E5, CMC-Na, and Na-alginate) and ODF are presented in Figure 9. 

The FT-IR spectrum of each ODF revealed an interaction between atenolol and film former. This interaction was observed from the disappearance of atenolol specific band, broadening of -OH stretching of atenolol, and the sifting of carbonyl groups absorption band of atenolol to the lower wavelength number. The interaction between atenolol and film former was predicted as hydrogen bonding. 

### 3.12. Molecular Docking Analysis

**Figure 10:**
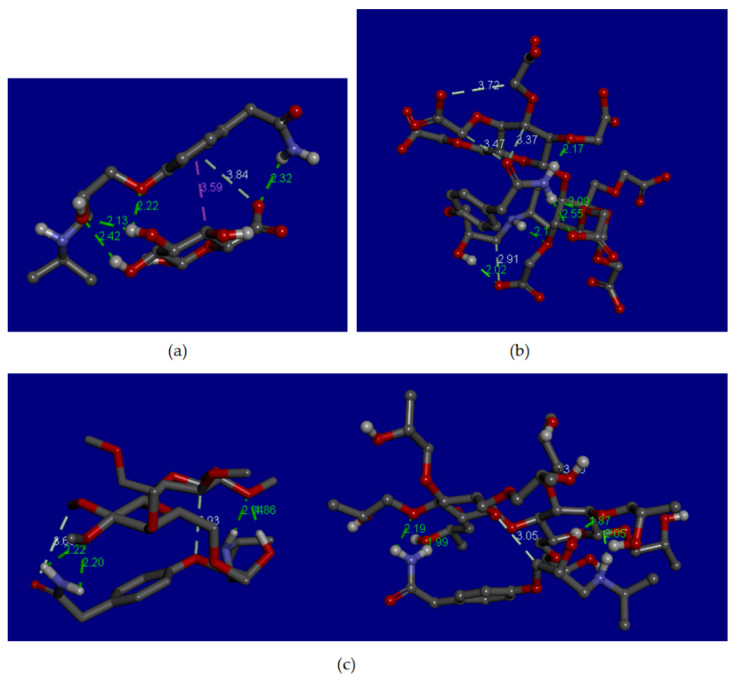
Molecular docking of (**a**) atenolol-sodium alginate; (**b**) atenolol-CMC-Na; and (**c**) atenolol and HPMC (left: I and right: II) indicates the presence of hydrogen and hydrophobic bonds between atenolol and the film former.

The free binding energy (∆G) in silico of atenolol-Na-alginate, and atenolol-CMC Na, were −2.22 and −4.80 kcal/mol, respectively. The ∆G in silico for atenolol-HPMC were −3.31 for HPMC I and −4.73 kcal/mol for HPMC II. The interaction of atenolol-Na alginate in silico showed the presence of hydrogen and hydrophobic bonds, as shown in Figure 10a. The hydrogen bonds are located between the H21 atom of atenolol and the O6 atom of the Na-alginate, the O1 atom of atenolol is bonded to H6 atom of Na-alginate, O2 atom of atenolol with two H atoms of Na-alginate (H6, and H8), with binding distances of 2.32, 2.20, 2.13, and 2.42 Å, respectively. The interaction of atenolol-CMC-Na in silico showed hydrogen bonds, as shown in Figure 10b. The hydrogen bonds were between H14 atom of atenolol and O12 of CMC-Na, H15 of atenolol and O4 of CMC-Na, H21 of atenolol and O8 of CMC-Na, and H22 of atenolol and O20 and O5 of CMC-Na with binding distances of 2.02, 2.20, 2.01, 2.55, and 2.17 Å, respectively. The interaction of atenolol-HPMC showed hydrogen bonds, as shown in Figure 10c. The HPMC had two values of ∆G in silico based on the 2D chemical structure depiction of HPMC (Pubchem® Hydroxypropylmethilcellulose compound) resulting in two different molecular structure. The molecular structure of the first HPMC has the IUPAC name of 2R,3R,4S,5R,6R)-2,3,4-trimethoxy-6-(methoxymethyl)-5-[(2S,3R,4S,5R,6R)-3,4,5-trimethoxy-6-(methoxymethyl)oxan-2-yl]oxyoxane which is named HPMC I, and the second HPMC has the IUPAC name of 1-[[(2R,3R,4S,5R,6S)-3,4,5-tris(2-hydroxypropoxy)-6-[(2R,3R,4S,5R,6R)-4,5,6-tris(2-hydroxypropoxy)-2-(2-hydroxypropoxymethyl)oxan-3-yl]oxyoxan-2-yl]methoxy]propan-2-ol which is named HPMC II. Therefore, in this study, docking was carried out on both structures. The interaction of atenolol-HPMC I showed hydrogen bonds between H14 and H5 atoms of atenolol with O8 atom of HPMC I, H21 and H22 atoms of atenolol with O6 atom of HPMC I. The two H atoms were interacted with one O atom with binding distances of 1.86, 2.04, 2.20, and 2.22 Å, respectively. Similar results were found in the interaction of atenolol-HPMC II that showed hydrogen bonds between H14 and H5 atoms of atenolol with O8 atom of HPMC II, H21 of atenolol and O13 of HPMC II, and H22 of atenolol with O6 atom of HPMC with binding distances of 1.87, 2.05, 1.99, and 2.19 Å, respectively.

## 4. Discussion

Atenolol is an antihypertension drug which reduces blood pressure primarily via the reduction of cardiac output through chronotropic and inotropic inhibitory mechanisms [15]. Oral dissolving film (ODF) is promising dosage forms to overcome oral route administration problem in specific individuals, especially in geriatric patient who suffer from hypertension [15]. ODF disintegrates and dissolves quickly upon contact with saliva in the oral cavity, accelerating the drug release. Some portion of the drug may be absorbed in the oral cavity, pharynx, and esophagus as the saliva passes down into the stomach. This process is called pre-gastric absorption. Pre-gastric absorption provides bioavailability improvement and rapid onset of action of the drug, including atenolol that undergoes first pass metabolism [5]. The type and concentration of polymer as a film former are critical parameters determining physical properties, disintegration time, and drug release. Film former used in ODF formulation must exhibit the flexible film, adequate mechanical properties, good wetting, and spreading ability. The film forming capacity of the polymer is described as the ability of a polymer to form a film that can be easily exfoliated from the casting surface without damage or torn [5]. The hydrophilic polymer is preferable to be used in the formulation of ODF as it exhibits sufficient film- forming and swelling capacity that leads to the rapid disintegration of ODF [12]. In this study ODF of atenolol was developed using three different hydrophilic polymers as film former, including HPMC E5, CMC-Na, and Na-alginate. Various concentration of glycerin was used to compensate film former concentration to maintain physical properties of ODF such as thickness and tensile strength and as a wetting agent.

The prepared films were homogenous, yellowish in color, and flexible. HPMC as a film former produced a smooth film surface, while CMC-Na and Na -alginate exhibited a gritty film texture. HPMC E5 showed better film forming capacity and a smooth and transparent appearance [19]. The gritty texture of ODF, when using CMC-Na and Na-alginate as film former, was predicted due to inconsistency of polymer thickness and bubble entrapment during the casting process [20]. Crystallization of atenolol in the film matrix was also possible to produce gritty texture in ODF with CMC-Na and Na –alginate polymers. Crystallization of the drug possibility occurs because the viscosity of matrix composition was not adequate to prevent drug sedimentation [21]. 

The thickness of ODF was evaluated with a screw gauge in order to evaluate the film homogeneity and the reproducibility of the preparation method. The thickness uniformity is also directly associated with the dose uniformity. The results exhibited that these polymers (HPMC E5, CMC-Na, and Na-alginate) are suitable for producing a good film thickness uniformity. The film thickness of ODF prepared in this study was in the range of 0.116 mm to 0.233 mm, so that the ODF of atenolol provides suitable film thickness (<0.3 mm) [15]. ODF was cut to size 3 × 2 cm film, then the weight of ODF was evaluated. All prepared formulas exhibited an excellent uniformity of weight with low deviation among manufacturing replications. The increased of film former concentration resulted in the increase of ODF thickness, which is implicated in an increase in the weight of the formulated ODF [22]. Atenolol content in ODF was analyzed using UPLC instrument. The results showed that the drug content is relatively high in all formulas. This phenomenon is related to the thickness homogeneity of ODF, resulting from pouring process in the solvent casting method. Homogeneity of thickness is an essential physical parameter in ODF evaluation as it has substantial implication for the precision of dose distribution of ODF [23]. An increased in the ODF thickness reflects an increase in drug content and variety of mass [22]. ODF, when using CMC-Na as a film former (F3 and F4), exhibited wider variation of film thickness compared to the other formulas. This condition probably occurred due to the more viscous casting mass of ODF when using CMC-Na as a film former. More viscous solutions are disadvantages because of de-aeration problem and non-homogeneity distribution on a casting plate [21]. 

ODF of atenolol prepared using HPMC E5 and CMC-Na as film former presents good folding endurance (>300 times) [13]. These results indicated that those ODFs are tough and flexible. Nevertheless, sodium alginate produced ODF with poor folding endurance. This characteristic in line with the previous study, which revealed that Na- alginate exhibited brittle film properties, possibly due to the stiffness gel structure. The increase of sodium alginate concentration in ODF formulation produces less flexible film properties [24]. From the results of mechanical properties, HPMC exhibited superior film forming ability, physical appearance, and flexibility. 

The swelling property is an essential parameter in ODF formulation. The swelling index is a parameter to describe ODF swelling property when in contact with an aqueous medium [25]. Hydrophilic film former in ODF needs to take up water, swell, and disintegrate rapidly in order to release the drug. The swelling index indicates the ability of polymer to absorb water. The extent of swelling in polymers is a resultant of the free energy of mixing and the elastic refractile force, which opposes the deformation [26]. The high values of swelling index indicate that the polymer can be easily penetrated by water molecule [27]. ODF prepared using CMC-Na as a film former showed the highest value of swelling index compared to the others. This phenomenon occurred because CMC-Na has high swelling properties. Hence HPMC and sodium alginate have moderate swelling properties [28]. The swelling rate of CMC-Na hydrogel was very high in the initial period until reaching the equilibrium of swelling. The pH of ODF was around neutral pH (pH = 7), hence CMC-Na exhibited optimum swelling in this situation. The result of this evaluation also revealed that the increased concentration of each film former did not reveal a significant difference in the swelling index of ODF.

A disintegration time test in artificial saliva was carried out to predict the ability of ODF to disintegrate into fragments in the oral cavity. The disintegration time of ODF prepared in this study ranged from 13.26 to 50.46 s. All of the film formers produced a suitable disintegration time of ODF, which was less than 60 s [20]. The result also revealed that increasing the concentration of the film former also increased the time needed for ODF to disintegrate. The concentration of the film former influenced the viscosity of the film matrix. The viscosity of the film matrix increased with the increase in the concentration of film former, therefore lengthen the disintegration time of ODF [29]. Na-alginate produced a significantly faster disintegration time of ODF (F5 and F6) compared to the other four formulas (*p* < 0.05). These results revealed that film thickness and swelling index significantly influenced the ODF disintegration time. The ODF of atenolol prepared using Na- alginate as a film former afforded the lowest film thickness and moderate swelling property. Therefore, these characteristics leads to a faster disintegration time of F5 and F6. ODF with CMC-Na as a film former showed the highest swelling index but exhibited the slowest disintegration time compare to the other film formers. This is due to the great swelling behavior and entrapment of water molecules in the polymer matrix resulting in gel formation, making the disintegration time slower [30]. 

ODFs of atenolol is required to release the drug in specific amount of the predetermined time limit. The amount of drug dissolved from ODF prepared using HPMC E5 as a film former, was significantly different from CMC-Na and Na-alginate (*p* < 0.05). In-vitro drug dissolution study revealed that HPMC E5 as a film former produced the highest percentage of drug dissolved in 30 min. HPMC exhibited a swelling-erosion mechanism of drug release within a film matrix. The previous study also revealed that HPMC induced rapid swelling and erosion. This mechanism proposes a rapid dissolution of the drug from the HPMC film matrix, despite the shortest disintegration time shown by Na-alginate as film former [31]. Na-alginate showed more retardation of drug release due to excessive swelling index than HPMC as film former [30]. Furthermore, as the concentration of film former increased, the drug release decreased due to the thickening of drug-film layer around the ODF. The increased of film former concentration also increased the wetting and dissolving time of ODF matrix [13].

The surface pH of ODF was evaluated to predict its possible effect on the mucous membrane of the mouth during the use of this dosage form. In the fact, acidic and basic oral formulation can cause an inflammatory reaction of the oral mucosa. Therefore, ODF formulation with neutral pH was intended in this study. The pH measurement results on the surface of the six ODFs in this study exhibited neutral pH, ranged from 6.94 to 7.4. These results indicated that ODFs would not irritate the oral mucosa and are acceptable to the patients. 

The particle shape and morphology of the ODFs were observed by a scanning electron microscope. Microscopic images of ODF revealed that formulas with HPMC E5 (F1 and F2) as a film former were gritty in texture, contained small pores, and the drug crystal was deposited in small particles on the surface of ODFs. The small pores in these ODFs indicate that water molecules can penetrate the film’s structure and shorten the disintegration time [32]. These findings supported the phenomenon that HPMC-based ODFs (F1 and F2) in this study are characterized by fast hydration, rapid disintegration, and quick dissolving of the drugs. However, ODF formulas with CMC-Na (F3 and F4) as a film former exposed high number of small pores and rough surfaces. The drug should dissolve in the film, but some portions of the drug was crystallized and adhered to the surface of ODFs. The drug crystal on the surface of ODF prepared using CMC Na was significantly larger than the ODFs prepared using the other formulas. This phenomenon was predicted as the excessive recrystallization of the drug in the film surface, indicating the matrix system was not strong enough to prevent drug sedimentation and crystallization [32]. Meanwhile, sodium alginate as a film former produced a rough, porous structure and widely open pores. Thus, the disintegration time of sodium alginate based ODFs (F5 and F6) was significantly faster than the other film formers. The observed drugs were homogenously distributed in the matrix cell wall of these formulas. In a further study, the drug deposition and distribution in the film matrix must be optimized to ensure the uniformity content of the drug. The inclusion complex of the drug using cyclodextrin or particle size reduction approach can be utilized to enhance content uniformity in each film [33]. 

The FT-IR spectroscopy study was conducted to analyze functional group and its vibration characteristic in ODF formulations. This analysis can predict the possible interaction between the drug and film former in ODFs. FT-IR spectrum of atenolol displayed a specific absorption band at 3400 cm^−1^ and 3100 cm^−1^ corresponding to the N-H group. The specific absorption band of atenolol at 1895 cm^−1^ represented the C=O bond. Hence absorption bands at 1660.41 cm^−1^ revealed the existence of C=O-N group. From the spectrum of ODFs, a slightly difference absorption band of -NH group of atenolol was observed. The absence or shifting of the carbonyl groups absorption band of atenolol was observed in ODFs spectrum. The broadening absorption band of -OH group in film former was also reflected. These results indicated the hydrogen bonding interaction between atenolol and film former [33].

Molecular docking analysis was also conducted in this study to predict and explain the results of the experimental study [34]. The affinity of the drug and excipient in silico was analyzed by the ∆G in silico. The higher affinity of the drug-excipient, the lower the ∆G in silico [35]. The ∆G in silico of atenolol-HPMC was the highest compared to atenolol-Na-alginate and atenolol-CMC Na. The results supported the experimental study.

The 3-D visualization of atenolol-Na-alginate in silico showed hydrogen and hydrophobic bonds. These results supported FTIR spectra of atenolol-sodium alginate, indicating hydrogen bonds between -NH (amine group) of atenolol and –OH group of Na-alginate. The 3-D visualization of atenolol-CMC Na in silico showed hydrogen bonds. These results supported FTIR spectra of atenolol-CMC Na that indicated hydrogen bonding, involving-N–H (amine) group of atenolol and –OH group of carboxylic acids in CMC-Na. The other interaction was also predicted to occur between the –N–H (amine) group of atenolol and the C=O (carbonyl) group of CMC-Na. The 3-D visualization of atenolol-HPMC in silico showed hydrogen bonds. These results supported FTIR spectra of atenolol-HPMC that indicated hydrogen bond interaction involving the amino and carbonyl groups of atenolol with the hydrogen group of HPMC E5. The molecular docking results supported the FT-IR spectrum analysis and revealed the hydrogen bonding between atenolol and each film former. 

## 5. Conclusions

In this study, atenolol was prepared as oral dissolving films by a convenient solvent casting method. Six formulas were developed using three different film formers (HPMC E5, CMC-Na, and Na-alginate) and two different concentrations of each film former. FT-IR spectrum and molecular docking analysis revealed the presence of hydrogen bonding interaction between atenolol and film former. ODF formulas developed in this study exhibited rapid disintegration time and drug release due to the porous structure confirmed by SEM analysis. Satisfactory results were obtained from several physical parameters including film thickness, folding endurance, swelling index, and disintegration time. However, HPMC E5 showed better film forming ability, physical properties, drug content homogeneity, and drug release profile compared to the other film formers. Formula 1 prepared using 4% (*w*/*v*) HPMC E5 is the most preferred formula to be developed in the further study. 

## Figures and Tables

**Table 1:** Composition of different film casting solutions.

Component	F1 HPMC E5 4% (mg)	F2 HPMC E5 5% (mg)	F3 CMC-Na 3% (mg)	F4 CMC-Na 4% (mg)	F5 Na-Alginate 2.5% (mg)	F6 Na-Alginate 3% (mg)
Atenolol	25	25	25	25	25	25
HPMC E5	40	50	-	-	-	-
CMC-Na	-	-	30	40	-	-
Na-Alginate	-	-	-	-	25	30
Glycerin	8	10	3	4	2,5	3
Poloxamer 188	0.3	0.3	0.3	0.3	0.3	0.3
Citric Acid	2	2	2	2	2	2
Potassium Acesulfame	3	3	3	3	3	3
Methyl Paraben	0.05	0.05	0.05	0.05	0.05	0.05
Orange Flavor	1	1	1	1	1	1
Sunset Yellow FDC	0.01	0.01	0.01	0.01	0.01	0.01
Propylene Glycol	2	2	2	2	2	2
Purified Water	1 mL	1 mL	1 mL	1 mL	1 mL	1 mL
Total Weight of ODF	81.36	93.36	66.36	77.36	60.86	66.36

Note: HPMC E5 (hydroxypropyl methyl cellulose type E5), CMC-Na (sodium carboxymethyl cellulose), Na-Alginate (sodium alginate) were expressed as a percentage of purified water (%*w*/*v*). Each of these formulas was prepared for 1 film.

**Table 2:** Orally dissolving film thickness.

Parameter	F1	F2	F3	F4	F5	F6
Film thickness (mm)	0.233 ± 0.031	0.228 ± 0.040	0.185 ± 0.001	0.206 ± 0.002	0.116 ± 0.006	0.136 ± 0.008

The results were expressed as mean ± SD, *n* = 4.

**Table 3:** Disintegration time of ODFs.

Parameter	F1	F2	F3	F4	F5	F6
Disintegration time (second)	31.99 ± 1.60	42.11 ± 0.57	40.47 ± 0.04	50.46 ± 0.09	13.26 ± 0.11	14.47 ± 0.28

The results were expressed as mean ± SD, *n* = 4.

## Data Availability

Not applicable.
